# Point-of-care ultrasonography for verification of central venous catheter placement in cats and dogs

**DOI:** 10.3389/fvets.2026.1745707

**Published:** 2026-02-25

**Authors:** Barbara Bruno, Andrea Degiovanni, Paolo Savarino, Antonio Borrelli, Alberto Tarducci, Silvia Rallo, Arianna Figini, Cristiana Maurella, Renato Zanatta

**Affiliations:** 1 Department of Veterinary Science, University of Turin, Torino, Italy; 2 Istituto Zooprofilattico Sperimentale del Piemonte, Liguria e Valle d'Aosta, Torino, Italy

**Keywords:** feline, canine, intensive care units, jugular venous catheter, PoCUS, ultrasound

## Abstract

**Introduction:**

This prospective study aimed to investigate the use of ultrasonography for verifying central venous catheter placement in hospitalized cats and dogs, in comparison with radiographic assessment.

**Methods:**

The investigation was conducted on client-owned animals. The position of the central venous catheter was checked using both thoracic radiography and ultrasonography. Ultrasonographic examination was performed in three steps: two intercostal scans of the cranial thorax (transverse and longitudinal scans), to visualize the course of the cranial vena cava within the mediastinum, and one right parasternal scan (sub-costal bicaval view), to visualize both the cranial and caudal vena cava entering the right atrium.

**Results:**

A total of 15 animals (8 dogs and 7 cats) were included in this study. Radiographic evaluation confirmed correct device placement within the cranial vena cava in 15/15 animals, with catheter extension into the right atrium observed in 8/15 cases. Ultrasonographic assessment of the mediastinal region demonstrated good agreement with radiographic findings (94%; CI95%: 83%−100%), and the bicaval atrial view showed high reliability in identifying central venous catheter tip location (Kappa = 0.87; CI95%: 65%−100%).

**Discussion:**

Ultrasound appears to be a reliable, non-invasive method for evaluating central venous catheter position in dogs and cats, with diagnostic accuracy comparable to radiography and the advantage of avoiding ionizing radiation exposure.

## 1 Introduction

Central venous catheters (CVCs) are utilized in critically ill canine and feline patients, both in intensive care and during anesthetic procedures. Central venous catheter placement involves inserting a long, relatively large-bore catheter through the external jugular vein into the cranial vena cava. The device is useful for patient monitoring (e.g., advanced hemodynamic monitoring, measurement of central venous pressure or serial blood samples) and allows the administration of hyperosmolar fluids or irritating drugs (1).

The device is also employed in human patients; however, differences in CVC placement exists between humans and small animals due to anatomical variations (e.g., cannulation of subclavian or internal jugular veins) and malposition has been reported in up to 14% of human patients, resulting in inaccurate central venous pressure readings, thrombus formation, vessel occlusion, through the vessel and pain (2–5) Malpositions are related to catheter presence in venous vessel other than cranial vena cava (CrVC), advancement into the heart or vessel puncture with secondary hemothorax or pneumothorax (2–5).

In veterinary medicine, CVC malposition is also reported and the main described sites are internal thoracic vein, azygos vein, caudal vena cava catheterization, paravenous locations and perforations of large vessels or right atrium. In particular, placement in the right atrium is only indicated in cases of extracorporeal circulation, because of the risk of arrhythmia, atrial perforation and pericardial tamponade (6–8).

After the CVC placement, the ability to easily aspirate blood does not guarantee correct placement and a standard chest radiography is recommended to verify the device position and to rule out possible complications, and it is currently considered the gold standard (8, 9).

In recent years, the application of point-of-care ultrasound (POCUS) has made central line insertion safer in human medicine, and it seems to perform at least as well as radiography for confirming CVC placement and screening for complications (10, 11).

The use of POCUS also offers several advantages: bedside assessment, repeat evaluations, rapidity, cost-effectiveness, dynamic images and the avoidance of one major disadvantage of X-rays, namely the exposure of patients and medical staff to radiation. Although there is evidence supporting the utility of POCUS technique compared to radiography, potential limitations exist, such as patient obesity, concurrent lung disease, the presence of pacemaker implants or chest wall anomalies (12–15).

The use of ultrasound for establishing vascular access has also been signaled in a few veterinary reports, with successful and rapid vascular access achieved in dogs. Despite an initial learning curve, the procedure can be acquired and applied by individuals of varying experience levels (16–18).

In this prospective study, we aimed to investigate the use of ultrasound to visualize the central venous catheter into the cranial vena cava and/or into the right atrium in hospitalized cats and dogs, by comparing ultrasound and radiographic evaluations.

## 2 Materials and methods

This was a prospective investigation of client-owned cats and dogs. The study protocol was approved by the Institutional Ethics and Animal Welfare Committee (protocol no. 1128). The owners were informed of the study protocol and each signed an informed consent form accordingly.

Cats and dogs admitted to the intensive care unit of the Veterinary Teaching Hospital were included in the study if they required placement of a CVC due to long hospitalization, for serial blood sampling and/or for administration of infusion fluids or drugs. Intracranial hypertension, hemostatic disorders, thrombocytopenia, severe skin lesions or soft tissue trauma of the neck or thorax were considered exclusion criteria, as they prevent CVC placement or could affect ultrasound evaluation. In addition, inability to perform the X-ray (e.g. inability to move the animal due to cardio-vascular/respiratory instability), accidental removal of the device or death of the animal before the completion of the investigations, precluded inclusion of some patients in the study.

### 2.1 Central venous catheter

In all animals, multiple-lumen CVCs were placed, using the modified Seldinger technique and kits including radiopaque catheter, scalpel, introducer, guidewire, dilator and caps (Certofix, B-Braun). Two catheter sizes were used depending on the animal's size: the first catheter had a diameter of 4 Fr and a length of 13 cm (with two 22 G lumens) and was used in cats and small dogs (< 10 kg), while the second catheter had a diameter of 7 Fr and a length of 20 cm (with 16 G lumens) and was used in dogs weighing more than 10 kg. Standardization of catheter placement was not part of the study, and the technique of CVCs placement was performed at the physician's discretion.

With animals in left lateral recumbency, skin was shaved, scrubbed and a sterile surgical drape was applied. A scalpel blade was used to make a stab incision through the skin over the external right jugular vein to be catheterized. An assistant then digitally occluded the jugular vein at the level of the thoracic inlet during placement of the introducer catheter. The introducer catheter was advanced percutaneously through the stab incision in the skin and into the external jugular vein until a flashback of blood was observed in the catheter hub (9). The stylet was then removed and the guidewire was inserted through the introducer catheter into the jugular vein. The introducer catheter was removed while keeping the guidewire in place, and the dilator was inserted and withdrawn over the guidewire, followed by placement of the CVC. The guidewire was removed, allowing it to appear from one of the proximal catheter lumens. At this point, using an empty 5 ml syringe, the CVC is aspirated until it is completely filled with blood and then flushed with sterile heparinized saline.

Before suturing the catheter wings, its position was checked by obtaining at least a lateral thoracic radiograph, followed by ultrasonography.

The CVC was considered adequately positioned if: the catheter was superimposed on the cranial vena cava; the catheter tip was located between the first rib and the cranial border of the cardiac silhouette; there was no evidence of pleural effusion or pneumothorax (unless it was present before CVC insertion) (8, 9). The radiographic prerequisites that allow for the correct evaluation of the CVC position were that the cranial border of the heart was clearly delineated by pericardial fat, the soft tissue/pulmonary interface cranial to the cardiac silhouette was sharply defined and the cranial mediastinal width was normal (8).

The ultrasound examiners were blinded to the catheter location detected by X-ray (cranial vena cava or right atrium).

### 2.2 Ultrasonography

Ultrasound examinations (EPIQ Elite release 10.1, Philips) were performed by a veterinary expert in ultrasound (PS, with more than 20 years of experience and a second-level Master's degree in ultrasound and cardiology), assisted by another sonographer during examinations and evaluation of images and videos (RZ and AD; veterinarians with more than 20 years of experience and/or a second-level Master's degree in ultrasound). A machine equipped with high-frequency linear transducer (eL 18–4 mHz), a microconvex transducer (mC 12–3mHZ), and a phased array transducer (S 9–2 mHZ) was used.

**Figure 1:**
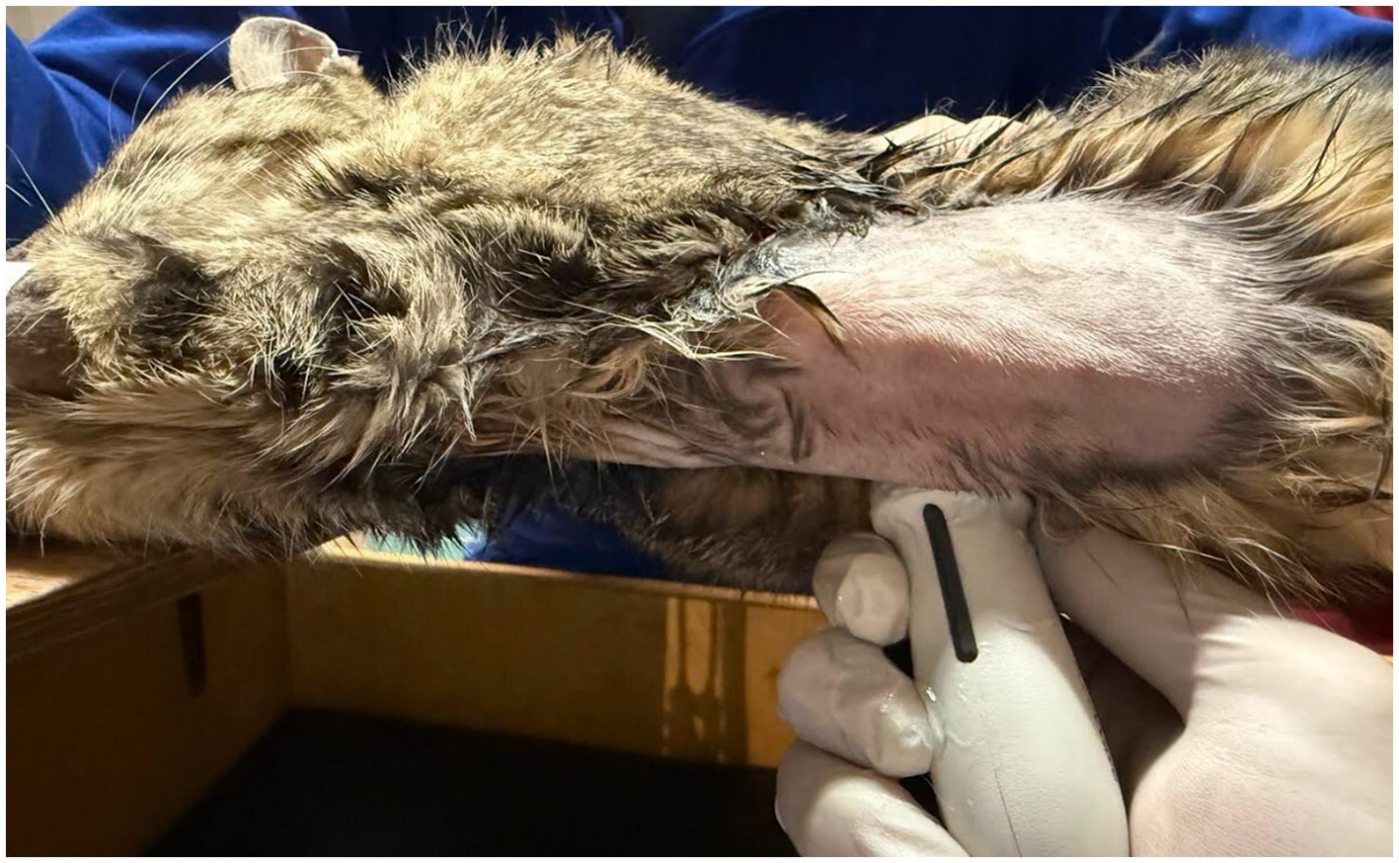
Sub-costal bicaval scan performed with phased array probe, to visualize the central venous catheter within cranial vena cava and right atrium.

**Figure 2:**
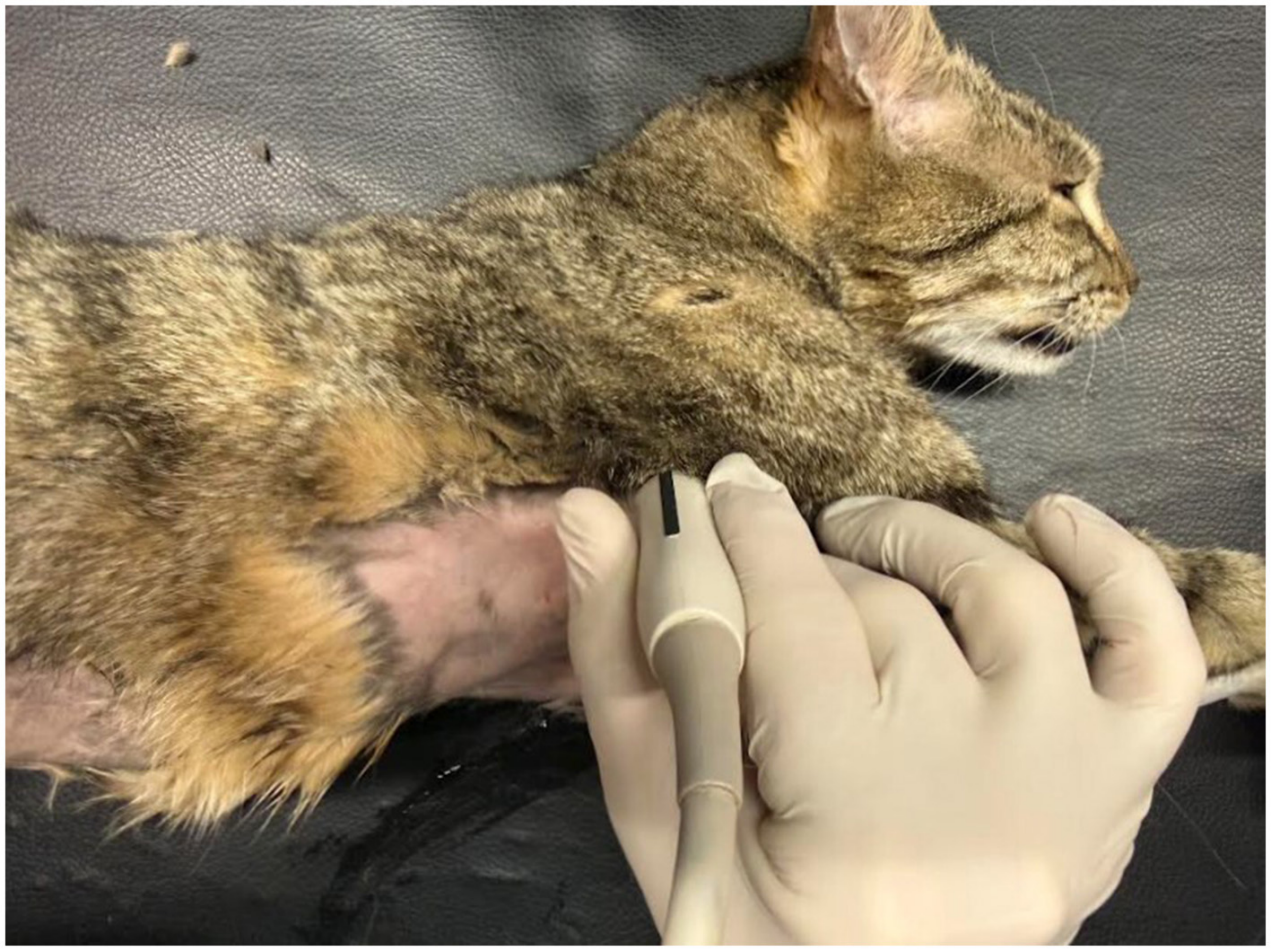
Transverse mediastinal scan of the right hemithorax with a microconvex transducer to identify the central venous catheter into the cranial vena cava.

The examination was performed in three steps on the right hemithorax: two intercostal scans at the cranial thorax (transverse and longitudinal scans), performed to visualize the course of the cranial vena cava (CrVC) in the mediastinum, using a microconvex transducer, and one transverse scan, called sub-costal bicaval view, to visualize both cranial and caudal vena cava entering into the right atrium, performed with a phased array probe (Figures 1, 2). Initially, another ultrasonographic scan was performed with a linear probe at the cervical site, between the insertion site of the catheter and the inlet of the thorax, to visualize the catheter within the external jugular vein, but since identifying the catheter in the cranial vena cava necessarily implied its presence in the jugular vein, the scan of the neck was abandoned.

**Figure 3:**
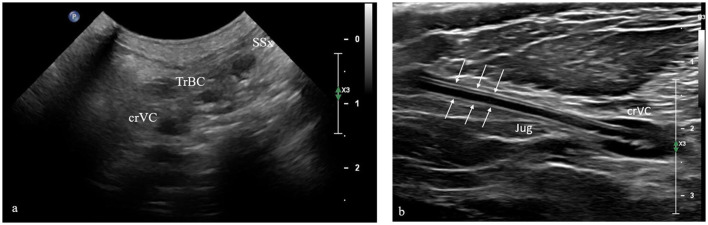
Transverse **(a)** and longitudinal **(b)** intercostal scans at the cranial thorax, performed to visualize the course of the cranial vena cava in the mediastinum. From the most superficial to the deepest, the subclavian artery, the brachiocephalic trunk, and the cranial vena cava were identified. In the longitudinal scan, the central venous catheter was visible as double parallel hyperechogenic lines with hypoechoic center (white arrows). The scans were performed with a microconvex probe. SSx, left subclavian artery; TrBC, brachiocephalic trunk; crVC, cranial vena cava; Jug, jugular vein.

Mediastinal scans were obtained by evaluating dogs and cats in lateral recumbency, moving forward the anterior leg to expose the axilla. At the 2^nd^-3^rd^ intercostal space, the probe was positioned perpendicular to the vertebral column, with the transducer marker oriented dorsally at the middle third of the ribs. The pleural line was followed cranially until a reduction of pulmonary tissue was observed, and three large regional vessels were identified, as well-defined, oval, anechoic structures. From the most superficial to the deepest, the subclavian artery, the brachiocephalic trunk, and the CrVC were identified (Figure 3a). To facilitate and confirm the identification of the vascular structures, color Doppler imaging was also used. The catheter was visualized in the CrVC as a rounded, hyperechoic structure with a hypoechoic center. At this point, the probe was rotated 90° degrees, without changing the inclination. This resulted in a longitudinal projection of the CrVC, which allowed the identification of the catheter as two parallel, hyperechoic lines inside the vessel (Figure 3b). In both scans described above, to confirm that the visualized structure was the CVC, we have measured its diameter (in longitudinal or transverse view), to compare it with the catheter French (one millimeter corresponds to 3 French).

**Figure 4:**
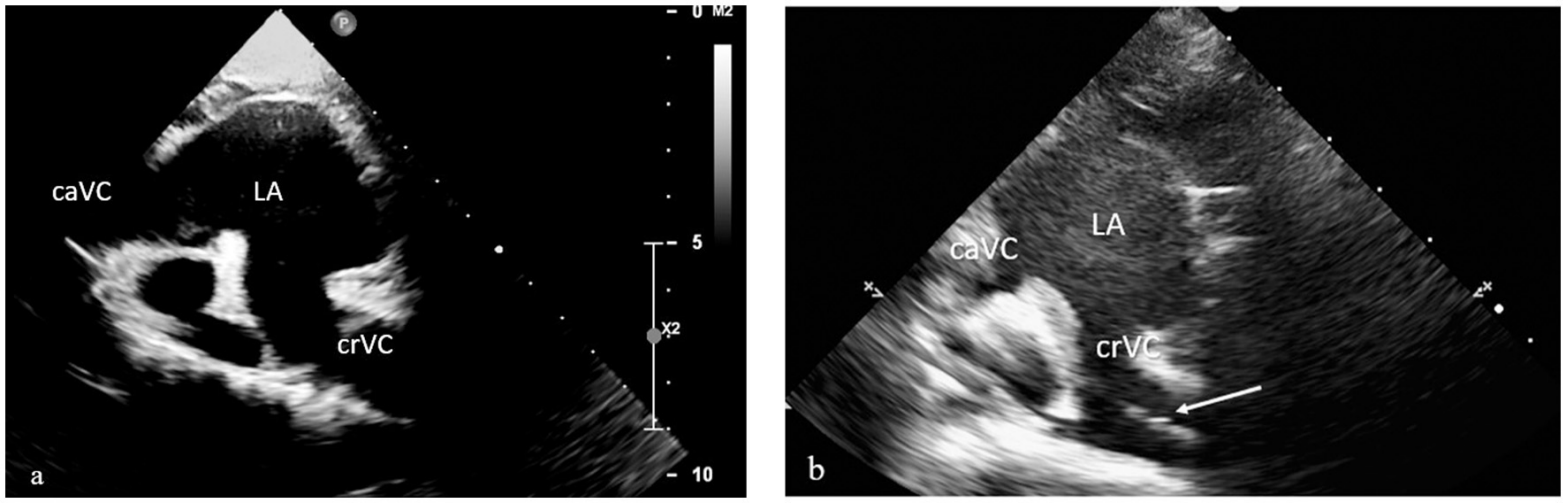
Sub-costal bicaval view, to visualize both cranial and caudal vena cava entering into the right atrium **(a)** and the central venous catheter within cranial vena cava **(b)**. The central venous catheter (white arrow in **b**) was identified as hyper-echogenic line within cranial vena cava. The scan was performed with a phased array transducer. RA, right atrium; caVC; caudal vena cava; CrVC, cranial vena cava.

The third scan is a bicaval atrial projection (a right parasternal scan of both venae cavae) obtained by positioning the probe on the right side of the animal's chest, toward the base of the heart, with the marker pointing toward the sternum (19). Starting from the right parasternal short-axis scan used to visualize the left atrium and aorta, the probe was tilted dorsally (toward the patient's vertebral column), with the probe marker directed cranially and rotated approximately 15 degree counterclockwise. This maneuver allowed a transverse view of the right atrium, with the inlet of the cranial and caudal vena cava, entering from the right side and left side of the atrium, respectively (Figure 4a). In this scan, the CVC can be visualized as hyperechoic lines, entering the right atrium from the CrVC (Figure 4b).

### 2.3 Statistical analysis

The required sample size was calculated to test the null hypothesis (H0): ρ_1_= ρ_2_, with α= 0.05 (two-sided) and a power = 80% (20). To assess the reliability between the two methods used, Cohen's kappa was calculated; in cases where all the samples showed the same result (i.e., all positives) and Cohen's kappa could not be computed, agreement was instead evaluated. Additionally, considering radiography as Gold Standard, the diagnostic accuracy of ultrasonography compared to radiographic evaluation was calculated in terms of sensitivity and specificity. All analyses were conducted using Stata version 18.1.

## 3 Results

A total of 19 animals were initially enrolled; however, four cases (one dog and three cats) were excluded due to the death or euthanasia that occurred before completion of radiographic and/or ultrasound examinations. Thus, 15 animals (eight dogs and seven cats) were ultimately included in the study.

The canine population consisted of three females (two spayed) and five males (one castrated). The median age was 7 years (min 3 and max 13 years) and the median body weight was 26.8 kg (min 6.2 and max 60 kg). Breeds were represented by two crossbreed dogs, and one representative of each of the following: German Shepherd, Rottweiler, Yorkshire Terrier, West Highland White Terrier, Labrador retriever and Lagotto Romagnolo. Underlying diseases included pancreatitis, abdominal carcinoma, chronic kidney disease, hyperosmolar diabetic syndrome, diabetic ketoacidosis, gastric dilation volvulus, peritonitis and hemorrhagic gastroenteritis (one dog per condition).

The feline population consisted of 6 neutered males and 1 spayed female. The median age was 10 years (min 1 and max 14 years) and the median body weight was 4 kg (min 2.3 and a max 6.2 kg). Breeds represented were European shorthairs (*n* = 5), Maine Coon (*n* = 1) and Oriental shorthairs (*n* = 1). Underlying diseases of the cats included diabetic ketoacidosis (*n* = 2), polytrauma, feline infectious peritonitis, urethral obstruction, intussusception and hepatic neoplasia (one cat per condition).

All CVCs were placed without complications, and no air or fluid accumulation was present following insertion of catheters. During hospitalization, inability to collect blood from the catheter were reported in 3 cases: in two animals, it was secondary to thrombus formation (one dog with a thrombus inside the right atrium, and one cat with a thrombus in the jugular vein), while in one dog, the difficulty to drawing blood was attributed to malposition of the catheter within the caudal vena cava.

Radiographs, which are considered the gold standard, allowed to visualize the catheter position in all animals (100%). The CVC tip reached the right atrium in 5/8 dogs (in one case the catheter extending beyond the cardiac silhouette and it was visualized above the liver) and in 3/7 cats. In the other 3 dogs and 5 cats, devices were detected within the CrVC by US and were correctly positioned proximal to the right atrium, as confirmed by radiography.

Visualization of the CVC in the cranial mediastinum was achieved with ultrasound scan in all dogs and in 6/7 cats (Table 1). In one cat, the catheter was not identified within the CrVC in the mediastinal view due to poor visualization of the three vessels, although the CVC was visible within the right atrium. On radiographic examination, the CVC was superimposed on the CrVC along the cervical segment, the mediastinum, and in the right atrium, confirming its position within the CrVC and its identification in the atrium by the ultrasound.

**Table 1:** Ultrasound and radiographic visualization of central venous catheters in dogs and cats.

**Animals N=15**	**XR**	**US**
CVC in CrVC	15	14
CVC in RA	8	9

CVC, central venous catheter; CrVC, cranial vena cava; RA, right atrium; XR, radiography; US, ultrasound.

The bicaval atrial ultrasonographic view allowed visualization of the catheter within the right atrium in 5/8 dogs and 4/7 cats (Table 1). As previously described, in one dog the catheter extended into the caudal vena cava and it was visible above the liver. In one cat, ultrasound evaluation revealed the presence of the catheter within the right atrium, but this was inconsistent with the radiographic findings, which showed the tip of the device located within the CrVC, before the atrium.

Agreement between the radiographic assessment and the ultrasonographic scan, performed at the mediastinum level, was 94% (CI95%: 83%−100%). Since CVCs were always visualized on radiographic studies (no negative radiographic results), Cohen's kappa could not be calculated. Accuracy of the bicaval scan compared with X-ray could not be calculated, as no negative cases are available (e.i. catheter seen in the atrium with the X-ray, but not with the ultrasound). The reliability between the X-ray and the bicaval atrial scan was Kappa=0.87 (CI95%: 65%−100%). The sensitivity in identifying the catheters that reached the atrium (and consequently identified by the X-ray, considered the gold standard) was 100% (CI95%: 69.2%−100%), while the specificity was 87.5% (CI95%: 47.3–99.7%).

## 4 Discussion

This study, conducted in cats and dogs, demonstrated that POCUS can visualize the CVC within the cranial vena cava and/or right atrium.

The analysis of the results obtained demonstrated that ultrasonographic assessment at the mediastinal level exhibited good agreement with radiographic findings, while the bicaval atrial view demonstrated high reliability. These findings suggest that the POCUS technique has a promising diagnostic performance for CVC localization.

A salient statistical finding was the inability to calculate Cohen's kappa coefficient. This outcome can be attributed to the 100% detection rate of CVCs via radiography in the present cohort, which consequently resulted in a paucity of negative marginal totals. While the Kappa statistic is the standard for reporting inter-rater reliability by correcting for chance agreement, its mathematical definition requires variance in both methods being compared. In this study, the ‘ceiling effect' of the radiographic gold standard resulted in a zero-variance scenario. Consequently, the high percentage of agreement (94%, 95% CI: 83%−98%) serves as the primary metric of clinical reliability, demonstrating that ultrasonography provides a nearly equivalent assessment to radiography in a real-world clinical setting with successful catheter visualization. Since there were no incorrect extra jugular positioning in this study, we do not know if the same agreement would be maintained in case of malposition.

Repeated bicaval atrial scans demonstrated values consistent with good repeatability, indicating that the same operator could reliably identify CVCs in the right atrium with satisfactory specificity.

Because ultrasound is frequently employed during hospitalization for other clinical evaluations, incorporating CVC assessment into routine POCUS appears feasible. The method is promising and it is rapid, non-invasive, avoids radiation exposure and moving the patient and enabling early detection of catheter misplacement, facilitating prompt repositioning and preventing complications. Correct CVC placement requires ensuring that the device is within the large vessels (e.g., jugular vein and cranial vena cava) without extending into the cardiac chambers. Except in case of hemodialysis catheters, placement of the tip within or beyond the right atrium required repositioning to prevent complication such as wall erosion or perforation, arrhythmias or inadvertent intracardiac infusion of chemical irritants (8). In our study, catheter repositioning was required in 8/15 animals (3/7 cats and 5/8 dogs), due to the CVC tip visualization in the right atrium or beyond. In particular, in one small dog the catheter was inserted to its full length, and the tip reached the liver. This finding could be explained by two factors: catheter excessive insertion depth or migration. Indeed, study protocol provided standardization for CVC monitoring by ultrasound, but not for device placement technique and we do not know if the length measurement from the insertion site to the caudal edge of the triceps muscle or first rib was performed. Furthermore, catheter tip migration was reported in neonatal human medicine, with higher frequency of migration (45% of cases) observed in catheters inserted via the upper extremities compared to those inserted more distal (20). It was speculated that, after placement, a polyurethane catheter can be softened by the body temperature and the blood stream may straighten its course, facilitating the advancement in the vessel (20). We do not know the time elapsed between CVC placement and suturing was unknown, and it is unclear whether this interval was sufficient to allow catheter migration; however the devices used were made of silicon, a particularly soft material.

In addition to locating the CVC, ultrasound could be useful in identifying causes of catheter malfunction, such as identifying thrombi (structure visualized inside the vessels or detected analyzing blood flow using color Doppler). Two thrombi were detected during the study: one in the right atrium (dog) and one in the jugular vein (cat). In the first case, fluid infusion was possible but blood aspiration was impaired due to thrombus formation. In the second case, catheter malfunction during blood sampling and fluid administration was explained by a thrombus that causes near-complete obstruction of the jugular vein, clearly visualized with cervical ultrasonography.

One discrepancy between ultrasound and radiography occurred in a cat, where poor cooperation and suboptimal scan quality led to misinterpretation of a cardiac structure as the catheter. Whereas radiography confirmed correct catheter placement with the tip in the CrVC. Although ultrasonographic identification of catheter position demonstrated a high success rate, a human study by Matsushima et al (21) reported one case in which a CVC tip was falsely identified as being in the right atrium. Potential risk factor associated with ultrasonography includes suboptimal image quality. As emphasized by previous studies, obtaining high-quality cardiac scan to visualize atriocaval junction is particularly important (21, 22). In another cat, mediastinal scan was considered negative for catheter identification, because the operator had doubts about the identification of the device into the cranial vena cava, due to poor vessels visualization. This result may have been influenced by projection issues, likely related to ribcage mobility and probe pressure on the thorax causing venous collapse; these factors can obscure catheter detection, if a hyperechoic structure, such as the catheter itself, approaches the vessel walls.

Several studies in human medicine provide evidence supporting the use of ultrasound for CVC insertion and confirmation of tip position, although slightly different protocols have been applied (10, 22–26). Procedures used across the different studies included US visualization of the catheter or the metallic guidewire, as well as the use of saline contrast (known as the “bubble test”), and thoracic scans and cardiac scans applied alone or in combination (10, 22–26). All studies agree that US had good sensitivity and specificity in identifying catheter misplacement compared with X-ray, and that the time needed to perform US scans was less than that required to obtain a chest X-ray (22–26). To combine methods and multiple scans allowed for better results, but each method carries some limitations: the bubble test and multiple scans protocols required extra equipment (agitated-saline syringes, different probes), at least one assistant and/or echocardiographic expertise (27).

Use of US to monitor the CVC placement has several advantages, in particular bedside assessment, cost-effectiveness and the reduction of patients and medical staff exposure to radiation (12–15). Furthermore, US appears to be more accurate than X-ray in identifying complications such as pneumothorax, haemothorax, pericardial tamponade and vascular thrombosis (10).

Despite previously reported evidence, radiographic confirmation of CVC position remains widely used, and US assessment is still inconsistently applied in human medicine, partly due to the absence of definitive recommendations in international guidelines (27, 28).

Few veterinary studies report the application of US for venous catheterization. In particular, ultrasound-guided vascular access has been evaluated as alternative to the standard procedure, with findings demonstrating that US allows successful and rapid vascular access in anesthetized dogs (16–18, 29). The application of the procedure by individuals with varying levels of experience was also assessed, observing that the protocol can be learned and applied after an initial learning curve and that confidence improves after the first attempt (16–18). Authors did not find studies evaluating the use of US for the assessment of CVC position.

Despite the promising findings of the present study, authors have identified some limitations. The sample size was small and included animals of different species and body conformations. Although the protocol was successfully applied to dogs ranging from 6.2 to 60 kg, further studies could be useful to evaluate whether animal size, thoracic conformations or body condition score influence ultrasound feasibility. Moreover, due to interspecies differences, comparative studies including a greater number of dogs and cats would help clarify species-specific advantages or limitations of the technique.

The ultrasound scan applied in our protocol requires skilled ultrasound operators and interpreters to be performed, especially the mediastinal scan. Before application of that ultrasound technique by emergency physicians, a training course with a learning phase should be planned. In human medicine, basic proficiency can be achieved with fewer than 10 h of training (15, 21, 30). Chamberlin et al. (16) detected a learning curve in veterinary operators performing ultrasound-guided jugular catheterization in dogs, suggesting that the technique can be easily learned by individuals of varying experience levels.

Furthermore, ultrasound evaluation could not always be a rapid assessment, but the duration of the procedure was not recorded in this study. Although transporting animals to the radiology unit is also time-consuming, it may be useful to compare the time spent to perform each procedure.

Another limitation could be having placed the animals in lateral positioning to perform the scans during the study, which might be dangerous or poorly tolerated by critically ill dogs and cats. Investigation the feasibility of previously described scans performed in sternal position and confirming their utility for CVC detection, could improve applicability in critical care settings.

## 5 Conclusion

The current study demonstrated that ultrasound has a good agreement (94%; CI95%: 83–100%) and high reliability (Kappa = 0.87; CI95%: 65–100%) to evaluate CVC position, compared to radiography. The technique offers the advantage of avoiding operator and patient exposure to ionizing radiation, aligns with current POCUS practices in emergency and intensive care units, and may allow earlier recognition of CVC malposition. Further studies are required to determine the time and training needed for emergency veterinarians, without advanced ultrasound expertise, to acquire the skills.

## Data Availability

The original contributions presented in the study are included in the article/supplementary material, further inquiries can be directed to the corresponding author.
